# Effect of pupil dilation on spherical and toric IOL calculations using a swept source OCT ocular biometer

**DOI:** 10.1007/s10792-026-03939-6

**Published:** 2026-02-09

**Authors:** Cameron McLintock, Samir Uprety, James McKelvie, Geraldine lee

**Affiliations:** 1 Department of Ophthalmology, Princess Alexandra Hospital, Brisbane, Queensland Australia; 2 Faculty of Medicine, The University of Queensland, Brisbane, Australia; 3 Vision for Life Institute, 1/1985 Logan Rd, Upper Mount Gravatt, Brisbane, Queensland Australia; 4 Department of Ophthalmology, University of Auckland, Auckland, New Zealand; 5 Faculty of Medicine and Health, The University of Sydney, Sydney, Australia

**Keywords:** Pupil dilation, Biometry, IOL, Anterion

## Abstract

**Background:**

We aim to evaluate the effect of pharmacological pupil dilation on spherical and toric intraocular lens (IOL) power calculations using biometric measurements from the Anterion optical biometer.

**Methods:**

This was a prospective observational study of adults undergoing cataract surgery. Pre- and post-dilation biometric measurements were obtained using the Anterion biometer. IOL power calculations, including both spherical and toric values, were performed using the following formulas: Barrett Universal II, Cooke K6, EVO, Hill-RBF, Hoffer® QST, Kane, and Pearl-DGS. Vector analysis was used to compare pre- and post-dilation corneal astigmatism and toric IOL magnitude and axis.

**Results:**

A total of 74 eyes from 37 patients (17 male, 20 female; mean age 52.4 ± 7.8 years) were analysed. Pupil dilation resulted in a statistically significant increase in reported central corneal thickness (CCT, *p* < 0.05), while other biometric parameters remained unaffected. Both spherical and toric IOL power calculations showed no significant changes across formulas following dilation. Vector analysis revealed that the centroid difference in corneal astigmatism and toric IOL magnitude ranged from 0.09 D to 0.05, and axis orientation differences ranged from 3° to 6°, indicating no systematic bias due to dilation.

Approximately 75% of eyes showed changes of < 0.50 D in spherical IOL power, while ~ 25% exceeded 0.50 D. For toric IOL power, ~ 90% of eyes showed changes < 0.50 D, with ~ 25% reaching or exceeding 0.50 D.

**Conclusion:**

Most eyes show minimal IOL power variation with pupil dilation; however, a subset may experience clinically relevant differences, particularly in toric IOL calculations.

**Supplementary Information:**

The online version contains supplementary material available at 10.1007/s10792-026-03939-6.

## Introduction

A key determinant of the refractive success of cataract surgery is accurate calculation of both spherical and toric intraocular lens (IOL) power and implantation axis. In busy clinical settings, it is not uncommon for clinicians to diagnose a visually significant cataract after a patient’s pupil has been pharmacologically dilated. This raises the question of whether biometry is accurate if performed on a dilated eye. Is it necessary to bring the patient back for a separate visit on a different day so that biometry can be performed on an undilated eye? Returning for repeat biometry on a separate day may not be practical for reasons such as transport difficulties or if the patient has a very busy work schedule.

Several studies have examined the influence of pupil dilation on ocular biometric parameters and have reported variable effects across the measured parameters using devices such as the IOLMaster, AL-Scan, and Lenstar [1–12]. However, to date, no studies have assessed the effect of pupil dilation on biometric parameters using the Heidelberg Anterion (Heidelberg Engineering GmbH), a relatively new optical biometer that employs swept-source optical coherence tomography (SS-OCT) technology. Given that inter-device discrepancies exist between the Anterion and other devices such as the IOLMaster, Galilei, and AL-Scan, it is important to determine the effect of pupil dilation on IOL power calculations performed with the Anterion.

Prior studies evaluating the effect of pupil dilation on IOL power have mainly utilised third generation (SRK/T, Hoffer Q) and fourth generation (Haigis, Holladay II) formula. Few studies have so far compared the dilation effect using fifth-generation formulas (Barrett Universal II, Hill-RBF, Olsen) [1, 5, 13].

Therefore, this study aims to investigate the effect of pupil dilation on ocular biometric parameters measured with the Anterion optical biometer. In particular, it aims to determine the impact of pupil dilation on intraocular lens calculations (IOL) power, both spherical and toric, using new generation formulas.

## Patients and methods

This prospective observational study pre and post-dilation biometric measurements from adult patients undergoing cataract surgery at the Princess Alexandra Hospital, Brisbane. All subjects provided written informed consent. The study adhered to the tenets of the Declaration of Helsinki, and ethical approval was granted by the Metro South Hospital Ethics Committee (LNR/2019/QMS/60356).

Prior to biometric evaluations, a comprehensive anterior and posterior segment examination was performed including macular and optic nerve OCT (Heidelberg Spectralis). Patients were eligible for the study if they were an adult patient referred for cataract surgery. Exclusion criteria included the presence of any co-existant ocular pathology, poor scan quality, recent contact lens wear, or a history of ocular surgery.

Ocular biometric measurements were obtained using the ANTERION (Heidelberg Engineering) by a single operator following the manufacturer’s guidelines. Measurement quality was assessed using the device’s internal acquisition parameters. If any parameter showed a failed or borderline status, the measurement was repeated up to three times. Scans that passed the quality assessment were included in the analysis, while scans that did not meet the manufacturer’s recommended criteria after repeats were excluded. Pre-dilation biometric measurement was performed, pupil dilation was achieved with 1% Tropicamide, and biometric parameters were remeasured 30 min later. Dilation was confirmed with a pupil diameter of ≥ 6 mm, assessed by the same operator. White-to-white (WTW) horizontal corneal diameter, pupil diameter, keratometry (Flat: K1 and Steep: K2), central corneal thickness (CCT), anterior chamber depth (ACD), lens thickness (LT), and axial length were obtained using the biometer. Intraocular lens (IOL) power calculations for the ZCB00 IOL (Johnson & Johnson Vision) were performed using formulas provided by the ESCRS online IOL calculator (https://iolcalculator.escrs.org), including Barrett Universal, Cooke K6, EVO, Hill-RBF, Hoffer®QST, Kane, and Pearl-DGS. For all formulas, the IOL power predicting a postoperative refraction closest to emmetropia was selected.

### Statistical analysis

Descriptive statistics (means and standard deviations) were calculated for continuous variables. All statistical analyses were performed using SPSS version 20.0, and plots were generated using GraphPad Prism version 10.0. Normality of the data was assessed using the Shapiro–Wilk test. Linear mixed-effect models were applied to assess the mean changes across ocular biometrics and IOL power between pre- and post-dilation, as well as to evaluate the differences between fellow eyes. Mixed models were fitted with a random intercept to adjust for within-subject variability and control for inter-eye correlation. Pairwise comparisons of pre- and post-dilation values from the mixed-effects model were adjusted for multiple testing using the Bonferroni correction. A *p*-value less than 0.05 was considered statistically significant. Based on previous studies, a minimum sample size of 36 was estimated to achieve 80% power at a 5% significance level for detecting a statistically significant difference [6, 7, 12]. Post‑hoc exploratory univariable logistic regression analyses were conducted to evaluate whether baseline biometric parameters were associated with the likelihood of a clinically significant (≥ 0.50 D) change in predicted IOL power following pupil dilation for each formula.

To assess whether the effect of pupil dilation on spherical and toric power was clinically significant, a threshold of 0.5D of sphere or cylinder was chosen as such a difference would result in a different IOL power being selected by the surgeon for implantation.

## Results

A total of 74 eyes (37 right and 37 left) from 37 patients (17 male, 20 female) were analysed. The mean patient age was 52.4 ± 7.8 years.

Table 1 shows the effect of pupil dilation on biometric parameters and IOL power calculations. Only CCT measurements showed a statistically significant difference between the undilated and dilated eyes (*p *< 0.05). Other measured parameters, including steep K2, steep K1, ACD, WTW, LT, and axial length, did not demonstrate significant changes. The effect of pupil dilation on IOL power calculations was also not statistically significant for any of the IOL formulas.

**Table 1:** Comparisons of biometric and IOL power measurements before and after pupil dilation

Pre dilation			Post dilation			
Parameter	Mean ± SD	Range	Mean ± SD	Range	MD	*P* value
Steep K2 (D)	44.28 ± 1.78	40.42, 49.47	44.33 ± 1.75	40.82, 48.95	−0.05	0.27
Flat K1 (D)	43.31 ± 1.41	39.51, 46.53	43.35 ± 1.40	39.80, 46.44	−0.04	0.36
CCT (μm)	535 ± 31	482, 624	537 ± 31	483, 625	−1.61	0.001
ACD (mm)	2.9 ± 0.6	1.5, 4.8	2.9 ± 0.6	1.6, 4.8	−0.06	0.19
WTW (mm)	11.9 ± 0.4	10.8, 13.0	11.9 ± 0.4	10.9, 12.9	−0.05	0.08
Lens thickness (mm)	4.5 ± 0.6	3.2, 6.0	4.4 ± 0.6	3.0, 6.0	0.09	0.09
Pupil Diameter (mm)	4.0 ± 0.9	2.7, 6.6	7.0 ± 1.3	6.9, 8.7	−2.99	< .001
Axial length (mm)	23.6 ± 1.1	21.6, 27.8	23.6 ± 1.1	21.6, 27.8	0.00	0.95
*IOL Power (D)*						
*Barrett*	20.86 ± 3.62	5.00, 27.50	20.77 ± 3.61	4.50, 27.50	0.09	0.33
*Cooke K6*	20.58 ± 3.52	5.00, 26.50	20.22 ± 4.20	5.00, 27.00	0.36	0.26
*EVO*	20.68 ± 3.77	2.50, 27.50	20.63 ± 3.68	3.50, 27.50	0.05	0.64
*Hill-RBF*	20.92 ± 3.58	4.50, 27.50	20.86 ± 3.57	4.50, 27.50	0.06	0.49
*Hoffer QST*	20.91 ± 3.88	2.50, 27.50	20.95 ± 3.88	2.50, 28.00	−0.04	0.74
*Kane*	20.89 ± 3.69	4.00, 27.50	20.99 ± 3.70	4.00, 28.00	−0.10	0.82
*Pearl DGS*	20.64 ± 3.55	5.00, 27.00	20.64 ± 3.48	5.00, 27.50	0.00	0.43

**Fig. 1:**
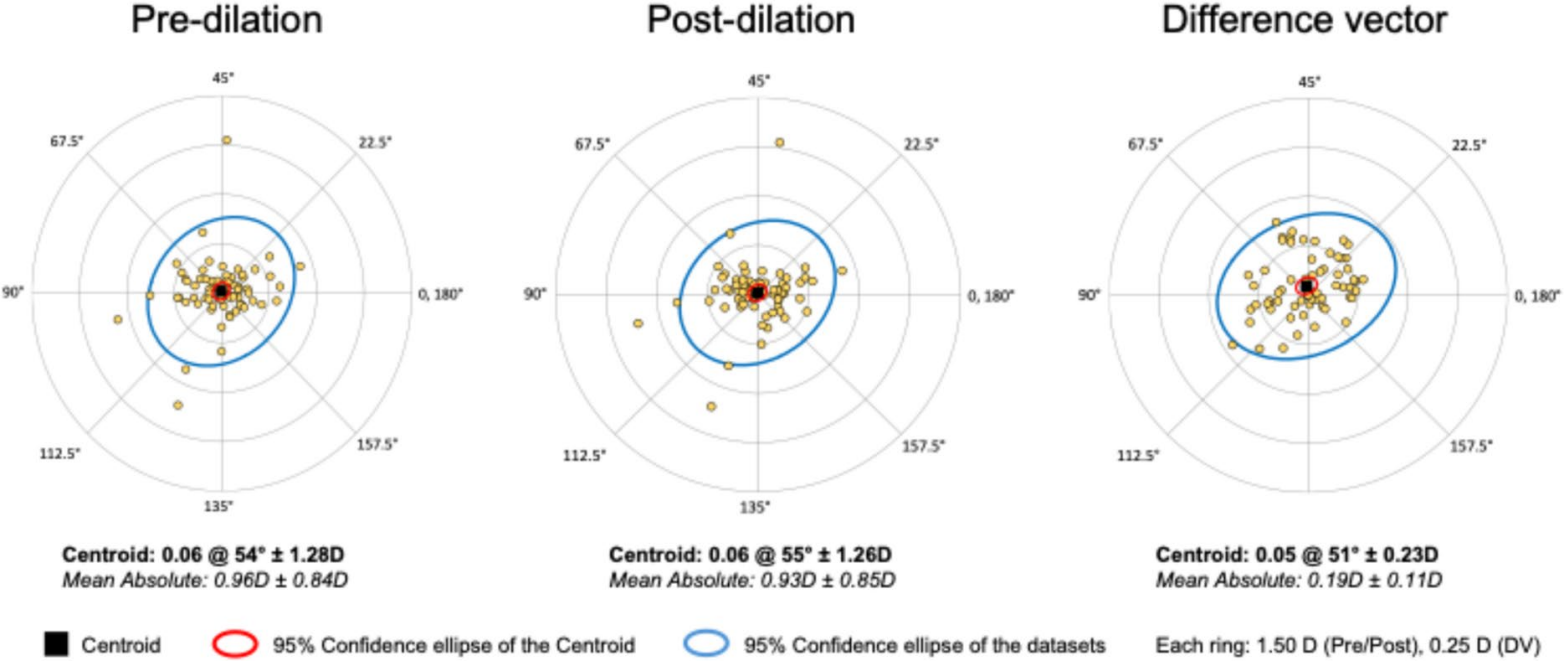
Double-angle plot demonstrating the effect of pupil dilation on corneal astigmatism

Vector analysis of corneal astigmatism before and after pupil dilation demonstrated minimal change in both magnitude and axis (Fig. 1). The centroid of pre-dilation astigmatism was 0.06 D at 54°, with a mean absolute astigmatism of 0.96 ± 0.84 D. Post-dilation, the centroid remained essentially unchanged at 0.06 D at 55°, with a mean absolute astigmatism of 0.93 ± 0.85 D. The difference vector, representing the change induced by dilation, had a centroid of 0.05 D at 51°, with a mean absolute magnitude of 0.19 ± 0.11 D. These findings indicate good stability of keratometric measurements under pharmacologically dilated conditions.

**Fig. 2:**
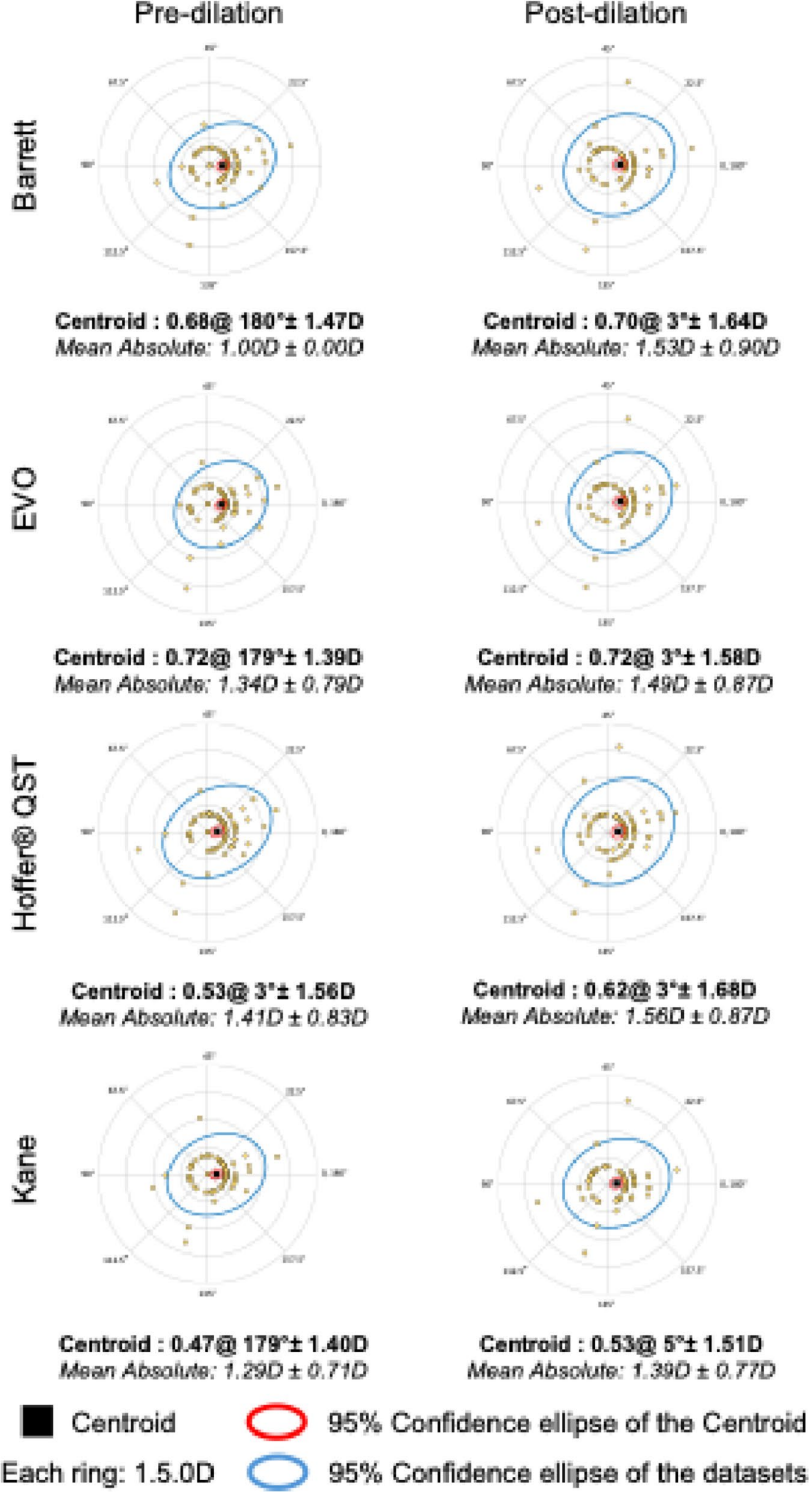
Double-angle plot demonstrating the effect of pupil dilation on toric IOL power calculation and axis orientation as estimated using the Barrett, EVO, Hoffer® QST, and Kane IOL formulas

Figure 2 demonstrates double-angle plots comparing toric IOL power and axis changes between pre- and post-dilation conditions for each toric IOL formula. The centroid vectors (averages) in each plot show minimal changes in magnitude (range: −0.02 to − 0.09 D) and axis orientation (3° to 6°), suggesting no systematic bias due to dilation. However, the mean absolute magnitude differences ranged from 0.1 to 0.5 D, indicating variability in toric IOL power as an effect of dilation.

**Fig. 3:**
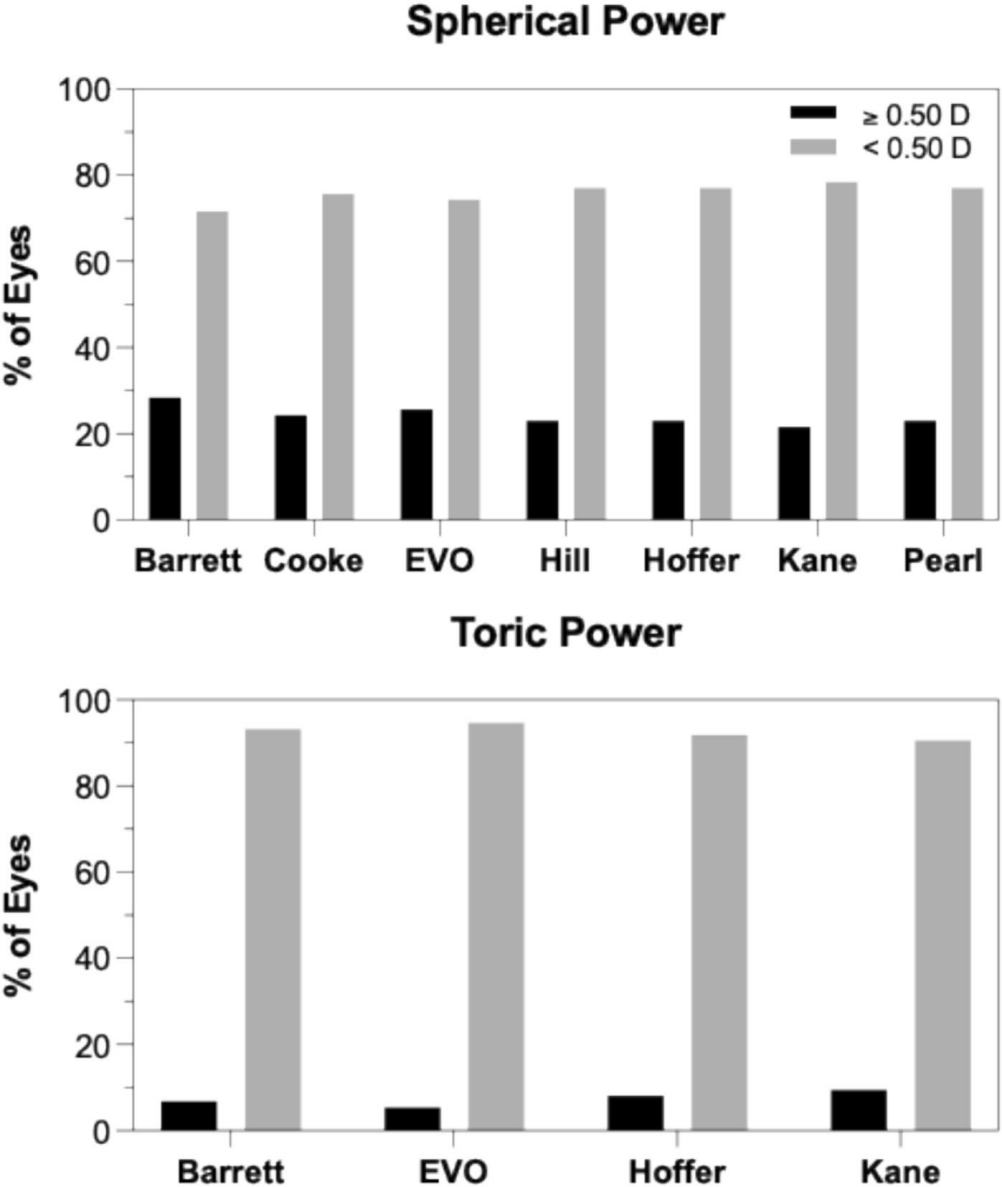
Percentage of eyes showing a clinically significant (0.5D) difference in sphere and toric power following pupil dilation, as estimated using various IOL formulas

Figure 3 illustrates the proportion of eyes showing changes in spherical and toric IOL power following pupil dilation. For toric power, more than 90% of eyes showed changes < 0.50 D, while nearly 10% exhibited changes ≥ 0.50 D across formulas. Similarly, for spherical power, nearly 75% of eyes had changes < 0.50 D, and 25% of eyes reached or exceeded the 0.50 D threshold. These results suggest that while most eyes experience minimal power variation after dilation, a notable subset shows potentially clinically relevant changes, particularly in toric IOL calculations.

In post‑hoc, per‑formula univariable logistic regression analyses using a ≥ 0.50 D change in predicted IOL power after dilation as the outcome, none of the baseline biometric parameters showed a statistically significant association with ≥ 0.50 D shifts for any formula. Across all formulas, lens thickness consistently demonstrated odds ratios of approximately 1.2, indicating a trend toward higher odds of a clinically meaningful IOL power change in eyes with thicker lenses, but this did not reach conventional significance (Supplementary Table 2).

## Discussion

This study suggests that the effect of pupil dilation on spherical IOL power estimation is IOL formula dependent. The Barrett Universal II formula demonstrated an IOL power difference greater than 0.50 D in 28% of eyes. Similarly, the EVO and Cooke formulas showed changes in approximately 25% of cases, while the Kane, Hill-RBF, Hoffer QST, and PEARL-DGS formulas exhibited such changes in around 23% of eyes. These findings indicate that the Kane formula may be less sensitive to dilation-induced biometric variations compared to the Barrett formula. In a similar study, Wang et al. (2018) also reported IOL power differences of ≥ 0.50 D in 48% of eyes using the Haigis and Olsen formulas in a paediatric population. In contrast, Arriola-Villalobos et al. (2013) and Bakbak et al. (2012) using the Lenstar LS, found that only 6 to 9% of eyes demonstrated changes greater than 0.50 D with the SRK/T and Holladay II formulas. Similarly, Can et al. (2016) using the AL-Scan biometer, reported that just 2.8% of eyes showed changes with SRK/T. These interstudy variations may potentially be attributed to differences in patient demographics, the use of age-specific formula such as Olsen, inter-device disagreement, and the varying sensitivity of individual formulas to changes in ocular biometry following dilation.

Regarding toric IOL power comparisons, the majority of eyes (> 90%) in our study showed minimal variation (≤ 0.50 D) following dilation, which is consistent with the observed dilation-induced changes in keratometric magnitude (93%). However, axis deviations greater than 10 degrees were observed in a formula-dependent manner. The Hoffer QST formula demonstrated axis variations exceeding 10 degrees in nearly 42% of eyes, whereas the Barrett Universal II, EVO, and Kane formulas showed axis deviations more closely aligned with the changes in keratometric axis induced by dilation, occurring in approximately 24% of eyes. These findings suggest that toric axis estimation is more susceptible to the effects of dilation than toric power estimation, and that the degree of susceptibility is formula dependent.

Although central corneal thickness (CCT) was the only anterior segment parameter that changed significantly in our study, most studies agree that ACD significantly increases post-dilation due to posterior movement of the iris–lens diaphragm and a slight reduction in lens thickness [1–6, 8, 11, 14–16]. Compared to these studies (mean difference range: from 0.001 mmm to 0.02 mm), we found a greater but non-significant difference in lens thickness, suggesting the variation reflects the changes in posterior movement of the lens. In addition, the absence of statistically significant changes in ACD and LT in our cohort likely reflects the small absolute magnitude of dilation-induced shifts in these parameters, which may lie below the detection threshold once inter-eye correlation and biological variability are accounted for, even though the direction of change is consistent with prior work.

The significant increase in CCT observed here is consistent with physiological responses to mydriasis, as mydriatic agents can induce transient corneal hydration changes or alter epithelial integrity, resulting in small increases in thickness. Importantly, while CCT changed significantly, its influence on IOL power calculation is minimal compared with AL, K, LT and ACD. Therefore, the observed differences in predicted IOL power are more likely attributable to the cumulative effect of small changes across multiple biometric parameters rather than to CCT alone.

These post dilation biometric changes have the potential to influence the prediction of effective lens position (ELP), an essential component in both spherical and toric IOL power calculations. The Hoffer QST formula relies primarily on ACD for ELP estimation and considers only the anterior corneal curvature (K), without incorporating compensation for posterior corneal astigmatism (PCA) [17]. In contrast, the Barrett Universal II formula employs a thick-lens vergence model with empirical refinements and integrates multiple biometric parameters, including ACD, lens thickness (LT), axial length (AL), and white-to-white (WTW), for more accurate ELP prediction. The EVO formula similarly accounts for ACD, LT, and CCT, and is optimized using machine learning. The Kane formula takes a hybrid approach, combining theoretical optics with artificial intelligence, using a comprehensive set of biometric variables to refine both ELP and axis predictions [18]. In addition to ELP modelling, differences in how formulas compensate for PCA further contribute to variability. Unlike Hoffer QST, which uses anterior keratometry alone, Barrett, EVO, and Kane incorporate PCA either through direct modelling or statistical estimation. Taken together, these differences in ELP estimation and PCA compensation likely explain the formula-specific variations in toric axis prediction observed in this study, despite identical keratometric inputs.

Apart from central corneal thickness (CCT) measurements, our study did not find any significant effect of pupil dilation on individual biometric parameters measured with the Anterion biometer. Studies using the IOLMaster, AL-Scan, or Lenstar biometers to assess the effect of dilation have reported inconsistent results across biometric parameters. Our findings regarding keratometric values were consistent with most studies, which have also reported no significant changes, supporting the stability of corneal curvature irrespective of pupil dilation [2–4, 6, 11, 14]. Only a few studies, such as those by Wang et al. (2018) and Bakbak et al. (2012) have reported significant changes in keratometric values following pharmacological pupil dilation. Similarly, our study and others examining the effect of dilation on white-to-white (WTW) diameter have resulted in inconsistent findings, suggesting no significant change. In contrast, Wang et al.[1] and Xi et al.[7] reported significant changes in WTW diameter, particularly in highly myopic eyes. It has been hypothesised that pharmacological dilation may alter ciliary muscle tone, indirectly affecting the scleral spur and peripheral cornea. Additionally, the shift in the image reference edge, from a lighter to a darker iris–limbus border becoming more prominent under dilation could contribute to inconsistent WTW measurements.[7]Our findings, along with other studies, show no significant change in axial length (AL) after pharmacological dilation. Although Wang et al. (2018) and Xi et al. (2022) observed small differences of about 0.01 mm, these variations are unlikely to affect intraocular lens (IOL) power calculations due to their minimal magnitude.

Limitations of our study include the relatively small sample size, though adequately powered for detecting mean differences in biometric variables, and the use of a single IOL model (ZCB00) which may restrict the generalisability of our findings to other IOL design. Although our study did not find a significant difference in biometric measurements pre- and post-dilation, this result could be influenced by the precision and repeatability characteristics of the Anterion device itself. Therefore, future studies should include multiple biometers to better understand the interaction between pupil dilation and instrument-specific measurement differences. Additionally, we did not assess postoperative refractive outcomes. As a result, we are unable to correlate any changes in preoperative biometry due to dilation with the eventual refractive success of the surgery.

In addition, some cataracts may become more clinically apparent after pharmacologic dilation, our study did not categorise patients based on cataract visibility pre- and post-dilation; therefore, we were unable to assess whether such eyes were more likely to demonstrate larger (> 0.50 D) differences in predicted IOL power. This represents an interesting consideration for future studies.

Although no baseline biometric parameters were significantly associated with clinically meaningful (≥ 0.50 D) IOL power shifts following dilation, a trend toward greater susceptibility in eyes with thicker crystalline lenses was observed. This finding may reflect subtle lens‑related biometric changes during dilation that are not fully captured by current models. Future studies with larger sample sizes or multivariable modeling may help identify specific biometric profiles at greater risk for dilation‑induced power prediction variability and refine formula performance in such eyes.

Despite the lack of postoperative refractive outcomes, our findings provide important insights into ANTERION measurements under dilated conditions. This is particularly relevant for understanding how instrument-specific factors may influence biometric measurements, especially given the known inter-device variability reported in the literature. Even small shifts in biometry can affect IOL selection, which is critical in eyes with borderline values or when planning premium IOL implantation. While previous studies have assessed dilation effects using other biometers, our study is among the first to systematically evaluate these changes using the ANTERION, including their impact on predicted IOL power. Future studies incorporating postoperative refractive outcomes and comparisons across multiple biometers are needed to confirm the clinical significance of these observations.

In conclusion, pharmacological pupil dilation had minimal effect on most ocular biometric parameters measured using the Anterion biometer. However, clinically significant changes in spherical IOL power predictions were observed in approximately 25% of cases, depending on the formula used. Toric power predictions showed clinically significant variation in only a minority of eyes, while toric axis predictions demonstrated formula-specific variability, with clinically significant deviations (> 10°) noted in a substantial proportion of cases, particularly with the Hoffer QST formula. To minimise dilation‑related error in IOL calculations, biometric measurements should preferentially be obtained under normal, undilated physiological conditions; however, surgeons may also consider patient‑specific visual demands (e.g. predominantly bright versus low‑light working conditions) during preoperative counselling, and discuss whether optimisation for the undilated state alone is appropriate or whether the potential impact of the dilated state on visual performance should also be taken into account.

## Supplementary Information

Below is the link to the electronic supplementary material.

Supplementary file1 (DOCX 27 KB)

## Data Availability

The datasets are available upon reasonable request from the corresponding author.
