# Supplementary material for: Effect of pupil dilation on spherical and toric IOL calculations using a swept source OCT ocular biometer
Source: Int Ophthalmol. 2026 Feb 9;46(1):106. doi: 10.1007/s10792-026-03939-6 (PMC12886208; doi:10.1007/s10792-026-03939-6)
Supplement: Supplementary file 1 — Supplementary file1 (DOCX 27 KB) [file 10792_2026_3939_MOESM1_ESM.docx]

Table 2 : Post‑hoc exploratory univariable logistic regression analyses for each IOL formula

| Outcome | Predictor | OR | Lower_CI | Upper_CI | p_value |
| --- | --- | --- | --- | --- | --- |
| Barrett IOL | Steep K2 | 1.3 | 0.972 | 1.736 | 0.077 |
| Barrett IOL | Flat K1 | 1.4 | 0.954 | 2.047 | 0.086 |
| Barrett IOL | CCT | 1.0 | 0.998 | 1.032 | 0.079 |
| Barrett IOL | ACD | 0.8 | 0.343 | 1.749 | 0.538 |
| Barrett IOL | WTW | 0.3 | 0.086 | 1.182 | 0.087 |
| Barrett IOL | Lens thickness | 1.2 | 0.893 | 5.311 | 0.087 |
| Barrett IOL | Pupil Diameter | 1.0 | 0.365 | 1.332 | 0.275 |
| Barrett IOL | Axial length | 0.9 | 0.539 | 1.429 | 0.599 |
| Cooke K6 IOL | Steep K2 | 0.9 | 0.675 | 1.246 | 0.580 |
| Cooke K6 IOL | Flat K1 | 1.0 | 0.685 | 1.458 | 0.997 |
| Cooke K6 IOL | CCT | 1.0 | 0.975 | 1.011 | 0.426 |
| Cooke K6 IOL | ACD | 0.8 | 0.135 | 1.076 | 0.068 |
| Cooke K6 IOL | WTW | 1.1 | 0.297 | 3.925 | 0.907 |
| Cooke K6 IOL | Lens thickness | 1.3 | 1.814 | 3.954 | 0.313 |
| Cooke K6 IOL | Pupil Diameter | 0.8 | 0.104 | 0.759 | 0.123 |
| Cooke K6 IOL | Axial length | 0.8 | 0.267 | 0.993 | 0.068 |
| EVO IOL | Steep K2 | 1.1 | 0.796 | 1.420 | 0.678 |
| EVO IOL | Flat K1 | 1.1 | 0.761 | 1.606 | 0.599 |
| EVO IOL | CCT | 1.0 | 0.983 | 1.016 | 0.936 |
| EVO IOL | ACD | 0.8 | 0.236 | 1.438 | 0.241 |
| EVO IOL | WTW | 0.8 | 0.090 | 1.301 | 0.115 |
| EVO IOL | Lens thickness | 1.3 | 1.944 | 3.621 | 0.219 |
| EVO IOL | Pupil Diameter | 1.0 | 0.232 | 1.074 | 0.076 |
| EVO IOL | Axial length | 0.8 | 0.477 | 1.363 | 0.421 |
| Hill-RBF IOL | Steep K2 | 0.9 | 0.676 | 1.262 | 0.619 |
| Hill-RBF IOL | Flat K1 | 0.9 | 0.612 | 1.326 | 0.597 |
| Hill-RBF IOL | CCT | 1.0 | 0.978 | 1.013 | 0.590 |
| Hill-RBF IOL | ACD | 0.9 | 0.113 | 1.011 | 0.052 |
| Hill-RBF IOL | WTW | 0.9 | 0.133 | 1.935 | 0.320 |
| Hill-RBF IOL | Lens thickness | 1.2 | 1.210 | 2.670 | 0.204 |
| Hill-RBF IOL | Pupil Diameter | 0.9 | 0.311 | 1.326 | 0.231 |
| Hill-RBF IOL | Axial length | 0.8 | 0.440 | 1.349 | 0.362 |
| Hoffer QST IOL | Steep K2 | 0.8 | 0.530 | 1.063 | 0.106 |
| Hoffer QST IOL | Flat K1 | 0.8 | 0.545 | 1.197 | 0.288 |
| Hoffer QST IOL | CCT | 1.0 | 0.969 | 1.006 | 0.195 |
| Hoffer QST IOL | ACD | 1.3 | 0.104 | 0.968 | 0.084 |
| Hoffer QST IOL | WTW | 1.3 | 0.344 | 4.817 | 0.708 |
| Hoffer QST IOL | Lens thickness | 1.3 | 1.594 | 2.643 | 0.058 |
| Hoffer QST IOL | Pupil Diameter | 0.8 | 0.138 | 0.888 | 0.071 |
| Hoffer QST IOL | Axial length | 0.9 | 0.350 | 1.183 | 0.156 |
| Kane IOL | Steep K2 | 1.0 | 0.710 | 1.329 | 0.857 |
| Kane IOL | Flat K1 | 0.9 | 0.618 | 1.360 | 0.665 |
| Kane IOL | CCT | 1.0 | 0.981 | 1.017 | 0.902 |
| Kane IOL | ACD | 1.0 | 0.210 | 1.481 | 0.241 |
| Kane IOL | WTW | 1.0 | 0.249 | 3.654 | 0.944 |
| Kane IOL | Lens thickness | 1.2 | 1.512 | 3.016 | 0.077 |
| Kane IOL | Pupil Diameter | 0.8 | 0.187 | 1.057 | 0.067 |
| Kane IOL | Axial length | 0.9 | 0.387 | 1.281 | 0.250 |
| Pearl DGS IOL | Steep K2 | 0.9 | 0.689 | 1.280 | 0.691 |
| Pearl DGS IOL | Flat K1 | 1.0 | 0.712 | 1.541 | 0.813 |
| Pearl DGS IOL | CCT | 1.0 | 0.979 | 1.014 | 0.711 |
| Pearl DGS IOL | ACD | 0.9 | 0.215 | 1.446 | 0.229 |
| Pearl DGS IOL | WTW | 0.9 | 0.164 | 2.331 | 0.477 |
| Pearl DGS IOL | Lens thickness | 1.2 | 1.487 | 3.759 | 0.079 |
| Pearl DGS IOL | Pupil Diameter | 0.8 | 0.197 | 1.048 | 0.064 |
| Pearl DGS IOL | Axial length | 0.9 | 0.240 | 0.952 | 0.058 |
